# Tratamento da Síndrome de Brugada: Um Caso Bem-sucedido de Ablação por Cateter de Radiofrequência

**DOI:** 10.36660/abc.20240501

**Published:** 2025-02-11

**Authors:** Mirella Esmanhotto Facin, Cristiano Faria Pisani, Luciana Sacilotto, Francisco Carlos da Costa Darrieux, Nelson Samesima, Maurício Ibrahim Scanavacca

**Affiliations:** 1 Instituto do Coração do Hospital das Clínicas Faculdade de Medicina Universidade de São Paulo São Paulo SP Brasil Instituto do Coração do Hospital das Clínicas da Faculdade de Medicina da Universidade de São Paulo, São Paulo, SP – Brasil

**Keywords:** Síndrome de Brugada, Eventos Arrítmicos Ameaçadores à Vida, Cardioversor Desfibrilador Implantável, Ablação por Cateter de Radiofrequência

## Introdução

A Síndrome de Brugada (SBr) é uma doença grave caracterizada por um padrão eletrocardiográfico distinto e um risco aumentado de arritmias cardíacas fatais.^1^ Tradicionalmente, seu tratamento limitava-se aos cardioversores desfibriladores implantáveis (CDIs).^2^ Nas últimas duas décadas, entretanto, a ablação por cateter de radiofrequência (ACRF) surgiu como uma opção terapêutica valiosa, principalmente para os pacientes considerados inelegíveis ao implante de CDI ou que já sofreram terapia apropriada pelo dispositivo.^3,4^ Todavia, registros sobre o sucesso desse procedimento em médio ou longo prazo ainda são escassos. Relatamos aqui o caso de um paciente sintomático com SBr que se submeteu à ACRF após sofrer um choque apropriado pelo CDI.

## Relato de caso

**Figura 1:**
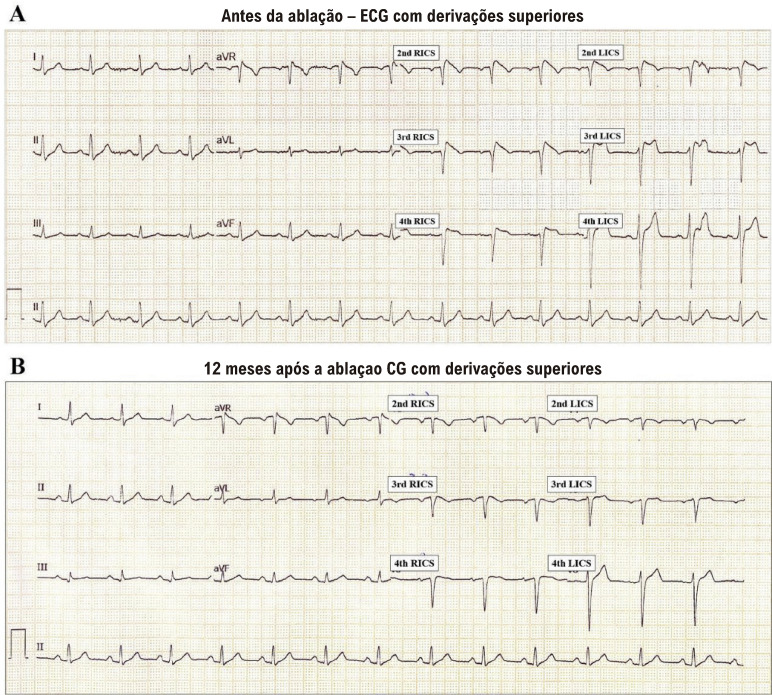
– Eletrocardiograma com derivações superiores antes da ablação (A) e 12 meses após a ablação (B).

Um homem branco de 51 anos foi encaminhado ao Ambulatótio de Arritmias Cardíacas do Instituto do Coração da Faculdade de Medicina da Universidade de São Paulo há 10 anos, devido à síncope. O paciente apresentara um único episódio de tontura e palpitação seguido de perda de consciência enquanto estava sentado, após o jantar, uma semana antes de sua primeira visita clínica. Com exceção de dislipidemia tratada com atorvastatina 40mg uma vez ao dia, a história clínica não apontava nenhum dado significativo. Não havia casos de doença cardíaca ou morte súbita entre os familiares. Não foi detectado qualquer achado relevante no exame físico. O eletrocardiograma (ECG) padrão (12 derivações) e o ECG com derivações superiores demonstraram um padrão Brugada (pBr) do tipo 1 (Figura 1A), que também foi evidente durante o monitoramento por Holter 24 horas e acentuado durante a fase de recuperação do teste ergométrico. A ressonância magnética cardíaca e a angiotomografia de artérias coronárias estavam normais, excluindo doença cardíaca estrutural associada.

O paciente recebeu o diagnóstico de SBr de alto risco com um escore de Shanghai estimado em no mínimo 5,5, uma vez que os resultados do teste genético ainda não estavam disponíveis. O incidente relatado de síncope possivelmente arritmogênica levou à implantação de um CDI como prevenção primária contra morte súbita cardíaca. Após 79 meses, o paciente sofreu um choque apropriado pelo CDI durante o sono após uma refeição pesada. Na tentativa de minimizar a recorrência de eventos arrímicos graves, a equipe de saúde recomendou a ACRF.

O procedimento foi realizado sob anestesia geral. O CDI foi reprogramado para o modo VVI a 40 bpm com desativação das terapias antitaquicardia. Em seguida, foram realizadas três punções na veia femoral direita, guiadas por ultrassonografia, e uma subxifoide conforme descrito por Sosa et al.^5^ Essa abordagem permitiu a inserção de um cateter Pentaray de mapeamento e um cateter quadripolar (3,5 mm) irrigado (SmartTouch SF) nas câmaras cardíacas direitas e no espaço epicárdico. Eletrodos de superfície foram usados para o monitoramento contínuo por ECG, e posicionados nos espaços intercostais superiores, para avaliar o trato de saída do ventrículo direito. O estudo eletrofisiológico convencional mostrou intervalos normais de condução, e a estimulação elétrica programada não induziu arritmias atriais ou ventriculares. O período refratário efetivo ventricular mais curto foi de 220 ms, no trato de saída do ventrículo direito, durante um ciclo de estimulação de 430 ms.

**Figura 2:**
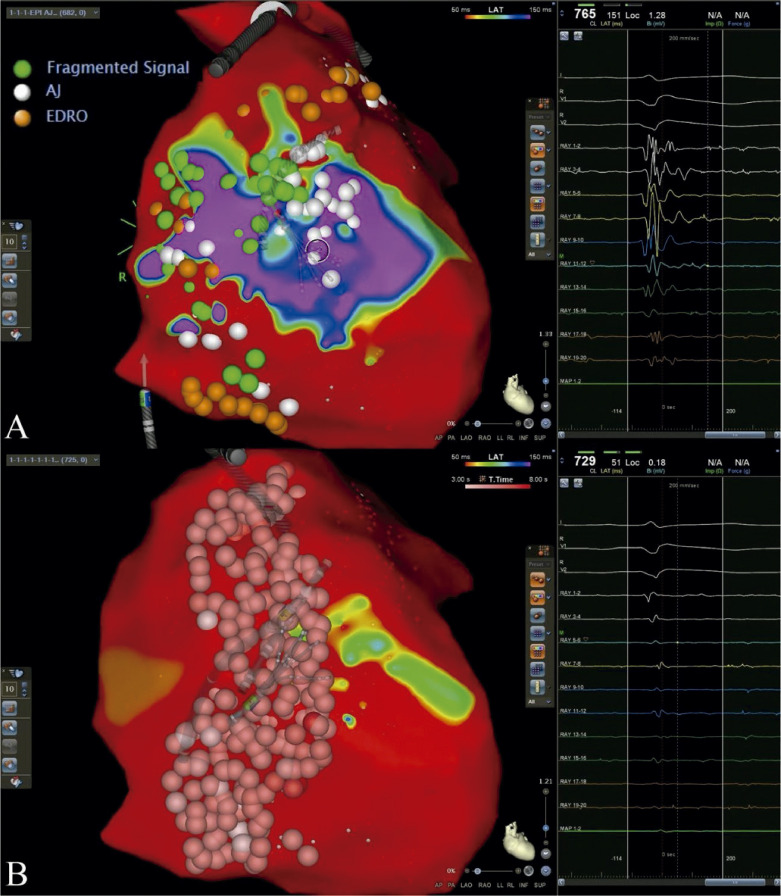
– Mapeamento eletroanatômico usando o sistema CARTO 3; vermelho mais curto que 50 ms, roxo mais longo que 150 ms. (A) Para o mapeamento, os locais de eletrocardiogramas anomais foram marcados durante o ritmo sinusal basal (verde), após infusão de ajmalina (branco), e após administração de edrofônio (laranja). (B) Locais de ablação na parede livre e na saída do ventrículo direito; não foram registrados eletrogramas de longa duração durante o remapeamento.

O mapeamento eletroanatômico da superfície endocárdica do ventrículo direito, realizado usando o sistema CARTO 3, não revelou áreas de cicatriz. No entanto, achados eletrofisiológicos anormais, caracterizados por potenciais tardios, fragmentados, e de baixa voltagem, foram localizados na superfície epicárdica da parede livre, estendendo-se para o trato da saída do ventrículo direito. A administração de ajmalina aumentou a extensão dos potenciais anormais, definindo assim os limites para a ablação. A ACRF foi aplicada por um cateter de ponta irrigada com sensor de contato, usando uma potência de 40W por 10 segundos em cada ponto de eletrograma epicárdico anormal. Em seguida, observou-se elevação convexa do segmento ST na superfície do ECG, com eletrodos precordiais posicionados bilateralmente no segundo, terceiro e quarto espaços intercostais. O tempo total do procedimento foi de 360 minutos, o tempo de fluoroscopia foi de 48 minutos (147 mGy) e o tempo de aplicação de radiofrequência foi de 40 minutos. O remapeamento imediato pós-ablação não demonstrou sinal anormal com ou sem administração de ajmalina (Figura 2).

A estimulação ventricular programada no ápice e no trato de saída do ventrículo direito, com ciclos basais de 600 e 430 ms, não induziu taquicardias ventriculares sustentadas. Após o procedimento, a aspiração pericárdica estava clara, sem sinais de perfuração ventricular, e os cateteres foram removidos sem complicações. As terapias do CDI foram reativadas, e o dispositivo reprogramado para sua configuração inicial.

Não houve intercorrências no período pós-operatório precoce, exceto por pericardite inflamatória leve, que melhorou rapidamente com administração oral de prednisona e colchicina. Três dias após a cirurgia, o paciente recebeu alta hospitalar. Visitas clínicas de seguimento foram agendadas 30, 90, e 180 dias após a ACRF e a cada seis meses depois.

Cada visita consistiu de uma avaliação abrangente, que incluiu anamnese, exame físico, interrogação do CDI, ECG de 12 derivações, ECG com derivações superiores (Figura 1B), teste ergométrico em esteira e monitoramento por Holter 24 horas. Doze meses após a ablação, o paciente foi submetido a um estudo eletrofisiológico convencional, que revelou ritmo sinusal e nenhuma evidência de pBr, nem no ECG padrão, nem no ECG com derivações superiores, mesmo após a administração de ajmalina. Intervalos basais encontravam-se dentro dos limites da normalidade, e o período refratário efetivo ventricular foi de 200ms tanto no trato de saída como no ápice do ventrículo direito, em um ciclo de estimulação de 430 ms. A estimulação ventricular programada até o S4 no trato de saída e no ápice do ventrículo direito não induziu arritmia.

O paciente permaneceu assintomático durante os 24 meses de acompanhamento, sem eventos arrítmicos detectados pelo aparelho. Testes complementares não invasivos demonstraram normalização do ECG e ausência de arritmias ventriculares malignas durante o período de acompanhamento.

## Discussão

Nós relatamos o caso de um paciente com SBr de alto risco que se submeteu à ACRF após sofrer uma intervenção apropriada pelo CDI. O paciente permaneceu assintomático e sem outras terapias pelo dispositivo, com achados eletrocardiográficos normalizados em todas as avaliações de acompanhamento no período de observação. Este caso destaca a potencial eficácia da ACRF em normalizar os padrões eletrocardiográficos, alcançando controle da arritmia e, assim, melhorando a qualidade de vida de pacientes com essa doença desafiadora.

A SBr é um problema de saúde importante, dada a sua alta prevalência em populações específicas e potencial letalidade, particularmente em homens de meia-idade, saudáveis sob outros aspectos. Indivíduos afetados tipicamente apresentam um pBr tipo 1 no ECG, com ondas J proeminentes, elevação do segmento ST do tipo côncava, e ondas T invertidas, observadas principalmente nas derivações precordiais direitas e (V1-V3), localizadas nos espaços intercostais padrões e superiores. O tipo e a duração dos eventos arrítmicos determinam a apresentação clínica, que pode variar desde ausência de sintomas até desmaios e morte súbita, principalmente em repouso ou outras condições vagotônicas.^2^

A estratificação de risco é crucial para decisões terapêuticas. Nosso paciente relatou um episódio de síncope possivelmente arritmogênica e apresentou um pBr tipo 1 espontâneo no ECG. O escore de Shanghai, um sistema usado para o diagnóstico e estratificação de risco da SBr, foi de pelo menos 5,5, o que indica uma alta probabilidade de se desenvolverem arritmias ventriculares.^6^ Além disso, sabe-se que os pacientes com sintomas arrítmicos, tais como síncope e taquicardia ventricular (TV) ou fibrilação ventricular (FV) são mais propensos a sofrerem eventos arrítmicos fatais.^2,6^ Diretrizes atuais recomendam que os pacientes sejam encaminhados para implantação de um CDI.^4^ Para os pacientes inelegíveis ao CDI ou que receberam terapias apropriadas do dispositivo, como nosso paciente, o tratamento medicamentoso ou a ACRF podem ser considerados, com base em consenso clínico bem estabelecido e avaliação de risco individualizado.^4^ No presente caso, devido à condição clínica do paciente e à falta de disponibilidade da quinidina, decidiu-se prosseguir com a ACRF. Estudos prévios mostraram resultados promissores com essa abordagem não farmacológica.^3,7,8^ O mapeamento eletroanatômico destacou o substrato arritmogênico da SBr: áreas dinâmicas de potenciais tardios, fragmentados e de baixa voltagem, localizados principalmente na superfície epicárdica do trato de saída do ventrículo direito.^3,8^ Manobras de provocação, tais como infusão de salina aquecida no espaço pericárdico ou administração de bloqueadores de canais de sódio como a ajmalina, podem acentuar essas áreas.^8,9^ Já foi comprovado que diferentes técnicas de ablação suprimem TV/FV recorrente e normalizam o padrão eletrocardiográfico em pacientes com SBr e alta carga arrítmica.^3,8,10^ Recentemente, Nademanee et al.^10^ relataram os desfechos da ablação do substrato epicárdico em 159 pacientes com SBr acompanhados por uma média de 48 meses.^10^ Segundo os autores, quatro de cada cinco pacientes não apresentaram recorrência de FV, o que aumentou para 96% após um segundo procedimento. Uma metanálise recente investigou 388 pacientes com SBr submetidos a ablação por meio de diferentes abordagens.^9^ Os autores observaram que menos de 10% dos pacientes tiveram recorrência de pBr tipo 1 no ECG, e menos de 20% apresentaram TV ou FV durante um período médio ponderado de 28 meses de acompanhamento após ACRF. A única complicação observada após a ACRF nesta série foi pericardite ou derrame pericárdico, ocorrendo em 9,3% dos pacientes, e controlados de maneira eficaz com medidas conservadoras.^9^

Este artigo apresenta o primeiro relato escrito de modificação do substrato arrítmico da síndrome de Brugada por ACRF via acesso epicárdico subxifoide no Brasil, demonstrando desfechos promissores na observação de médio prazo. Desde a intervenção, o monitoramento eletrocardiográfico mostrou, de maneira consistente, ausência de evidência de pBr e, o importante, ausência de outros eventos arrítmicos fatais ao longo do período de acompanhamento clínico de 24 meses. Independentemente da estratégia adotada, a ACRF é um tratamento preciso e direcionado que pode fornecer uma solução efetiva e segura para indivíduos com SBr. Contudo, o protocolo mais adequado para identificar os alvos da ablação e as melhores abordagens para a aplicação de radiofrequência ainda precisam ser definidos. Até o presente, existem poucos dados sobre o seguimento em longo prazo dos pacientes após a ablação, e não há evidência que corrobore o uso da ablação em pacientes assintomáticos. Mais pesquisas são necessárias para esclarecer o papel da ACRF no manejo de pacientes com síndrome de Brugada.
