# Supplementary material for: Tratamento da Síndrome de Brugada: Um Caso Bem-sucedido de Ablação por Cateter de Radiofrequência
Source: Arq Bras Cardiol. 2025 Feb 11;122(2):e20240501. [Article in Portuguese] doi: 10.36660/abc.20240501 (PMC11870120; doi:10.36660/abc.20240501)
Supplement: Translation

# Translation: Treating Brugada Syndrome: A Case of Successful Radiofrequency Catheter Ablation

**DOI:** 10.36660/abc.20240501i

**Authors:** Mirella Esmanhotto Facin, Cristiano Faria Pisani, Luciana Sacilotto, Francisco Carlos da Costa Darrieux, Nelson Samesima, Maurício Ibrahim Scanavacca

**Affiliations:** 1 Brazil Instituto do Coração do Hospital das Clínicas da Faculdade de Medicina da Universidade de São Paulo, São Paulo, SP – Brazil

**Keywords:** Brugada Syndrome, Life-threatening Arrhythmic Events, Implantable Cardioverter Defibrillator, Radiofrequency Catheter Ablation

## Introduction

Brugada syndrome (BrS) is a severe health condition characterized by a distinctive electrocardiographic pattern and an increased risk of life-threatening cardiac arrhythmias.^1^ Traditional therapy has been limited to implantable cardioverter defibrillators (ICD).^2^ In the past two decades, however, radiofrequency catheter ablation (RFCA) has emerged as a valuable treatment option, especially for those ineligible for ICD implantation or who have already undergone appropriate device therapy.^3,4^ Nevertheless, reports on medium to long-term success of this procedure are still scarce. We reported herein the case of a symptomatic patient with BrS who underwent RFCA after experiencing an appropriate ICD shock.

## Case Report

A 51-year-old white male was referred to the outpatient Cardiac Arrhythmia Unit at the Heart Institute (InCor) of the University of São Paulo Medical School due to syncope ten years ago. He experienced a single episode of dizziness and palpitation followed by loss of consciousness while seated after dinner a week before his first clinical visit. Except for dyslipidemia treated with Atorvastatin 40 mg once daily, his past medical history was unremarkable. There were no instances of heart disease or sudden death among his family members. No notable findings were detected during physical examination. The standard 12-lead and upper-lead electrocardiogram (ECG) demonstrated a spontaneous type 1 Brugada pattern (BrP) (Figure 1A), which was also evident during the 24hour-Holter monitoring and further accentuated during the recovery phase after the treadmill exercise test. Cardiac magnetic resonance imaging and coronary computed tomography angiography were unremarkable, effectively ruling out associated structural heart disease.

**Figure 1:** – Pre- (A) and post- (B) ablation electrocardiogram (ECG) with upper precordial leads.

The patient received a diagnosis of high-risk BrS with a Shanghai score of at least 5.5 since the results from genetic testing were pending. The reported incident of possible arrhythmic syncope prompted the implantation of an ICD as primary prevention against sudden cardiac death. Seventy-nine months later, the patient experienced an appropriate ICD shock during sleep following a heavy meal. Given the occurrence of this event, the healthcare team recommended RFCA to mitigate the risk of future arrhythmias.

The procedure was performed under general anesthesia. The ICD was reprogrammed to VVI mode at 40 bpm with anti-tachycardia therapies deactivated. Subsequently, three ultrasound-guided punctures in the right femoral vein and one fluoroscopy-guided subxiphoid approach, as described by Sosa et al.,^5^ were performed. This approach allowed the insertion of a Pentaray mapping catheter and an irrigated quadripolar 3.5 mm SmartTouch SF ablation catheter into the right cardiac chambers and epicardial space. Surface electrodes were used for continuous ECG monitoring, placed in the superior intercostal spaces to assess the right ventricular outflow tract (RVOT). Conventional electrophysiology study (EPS) yielded normal conduction intervals, and programmed electrical stimulation failed to induce any atrial or ventricular arrhythmias. The shortest ventricular effective refractory period (VERP) was 220 ms in the RVOT during a stimulation cycle of 430 ms.

Electroanatomical mapping of the right ventricular endocardial surface, performed using the CARTO 3 System, did not reveal any areas of scarring or abnormal potentials. However, abnormal electrophysiological findings, characterized by late, fragmented, and low-voltage potentials, were localized in the epicardial surface of the right ventricular free wall, extending into the outflow tract. Administration of ajmaline further increased the extent of abnormal potentials, thereby defining the boundaries for RFCA. RFCA was applied through an irrigated tip catheter with contact sensor using a power of 40W for 10 seconds at each point of abnormal epicardial electrograms. Subsequently, convex ST-segment elevation was observed in the surface ECG, with precordial electrodes placed bilaterally in the 2nd, 3rd, and 4th intercostal spaces. The total procedure time was 360 minutes, fluoroscopy time of 48 minutes (147 mGy) and radiofrequency application time was 40 minutes. Immediate post-ablation remapping demonstrated no abnormal signals with and without Ajmaline administration (Figure 2).

**Figure 2:** – CARTO 3 electroanatomical mapping; red shorter than 50ms, purple longer than 150ms. (A) For the mapping, the sites of abnormal electrograms were tagged during the baseline sinus rhythm (green tag), after ajmaline infusion (white tag), and after edrophonium (orange tag); (B) Ablation sites in the outflow and right ventricular free-wall; no long-duration electrograms were recorded on remapping.

Programmed ventricular stimulation at the apex of the right ventricle and RVOT, with baseline cycle lengths of 600 and 430 ms, failed to induce sustained ventricular tachycardias. After the procedure, pericardial aspiration was clear, without signs of ventricular perforation, and catheters were removed without complications. The ICD therapies were reactivated, and the device was reprogrammed to its original settings.

The early postoperative period was uneventful, except for mild inflammatory pericarditis, which resolved rapidly with oral prednisone and colchicine. Three days postoperatively, the patient was discharged from the hospital. Follow-up clinical visits were scheduled 30, 90, and 180 days after RFCA and every six months thereafter.

Each follow-up visit consisted of a comprehensive assessment, including history taking, physical examination, and evaluation of data on the ICD, standard 12-lead and superior lead ECG (Figure 1B), treadmill exercise testing, and 24-hour Holter monitoring. Twelve months post-ablation, the patient underwent an EPS, which revealed normal sinus rhythm and no evidence of the BrP, either in standard or upper-lead ECG, even after ajmaline administration. Basic intervals were within normal limits, and VERP measured was 200ms in both RVOT and right ventricular apex at a 430ms stimulation cycle. Programmed electrical stimulation up to S4 in the RVOT and right ventricular apex failed to induce any arrhythmias.

The patient remained asymptomatic throughout the 24-month follow-up period, with no arrhythmic events detected on device interrogation. Non-invasive complementary testing demonstrated normalization of the ECG and the absence of malignant ventricular arrhythmias throughout the follow-up period.

## Discussion

We report the case of a patient with high-risk BrS who underwent RFA after experiencing an appropriate ICD intervention. The patient has remained asymptomatic and free from further ICD therapies, with normalized electrocardiographic findings in all follow-up assessments throughout a 24-month observation period. This case highlights the potential efficacy of RFCA in normalizing the electrocardiographic pattern, achieving arrhythmia control, and, thus, improving the quality of life for patients with this challenging condition.

BrS remains a significant health concern due to its high prevalence in specific populations and potential lethality, particularly among middle-aged, otherwise healthy men. Afflicted individuals characteristically exhibit a type 1 BrP on the ECG, with prominent J waves, coved-type ST-segment elevation, and inverted T waves, most observed in the right precordial leads (V1-V3), placed in either standard or upper intercostal spaces. The type and duration of the arrhythmic events determine clinical presentation, which can range from no symptoms at all to fainting spells and sudden death, most commonly at rest or other vagotonic conditions.^2^

Risk stratification is crucial for treatment decisions. Our patient reported a potential arrhythmogenic syncope episode and displayed a spontaneous type 1 BrP on ECG. The Shanghai score, a system used for diagnosing and stratifying the risk of BrS, was found to be at least 5.5, which indicates a higher probability of developing critical ventricular arrhythmias.^6^ Additionally, it is widely known that patients with arrhythmic symptoms such as syncope and previously documented ventricular tachycardia (VT) or ventricular fibrillation (VF) are more likely to experience recurring life-threatening arrhythmic events.^2,6^ Current guidelines recommend these patients be referred for ICD placement.^4^ For individuals who have declined ICD implantation or experienced appropriate device therapies, such as our patient, drug therapy or RFCA may be considered based on established clinical consensus and individualized risk appraisal.^4^ In the present case, due to patient’s clinical condition and the unavailability of quinidine, it was decided to proceed with RFCA. Previous studies have shown promising results with this non-pharmacological approach.^3,7,8^ Electroanatomical mapping highlighted the arrhythmogenic substrate of BrS: dynamic areas of late, fragmented, and low-voltage potentials, primarily located in the epicardial surface of the RVOT.^3,8^ Provocation maneuvers, such as infusion of warm saline into the pericardial space or intravenous administration of sodium channel blockers like ajmaline, may accentuate these areas.^8,9^ Different ablation techniques have already been proven to suppress recurrent VT/VF and normalize the electrocardiographic pattern in patients with BrS and high arrhythmic burden.^3,8-10^ Recently, Nademanee et al.^10^ reported the outcomes of epicardial substrate ablation in 159 BrS patients followed for an average of 48 months.^10^ According to the authors, four of every five patients were free from post-ablation VF recurrence, which increased to 96% after a second procedure. A recently published meta-analysis investigated 388 patients with BrS who underwent RCA through different approaches.^9^ The authors found that less than 10% of patients experienced a recurrence of type 1 Brugada ECG pattern, and less than 20% experienced VT or VF during a mean weighted follow-up period of 28 months after RFCA. The sole complication observed following RFCA in this series was pericarditis or pericardial effusion, occurring in 9.3% of patients, which was managed effectively with conservative measures.^9^

This manuscript presents the first written report on arrhythmic substrate modification of Brugada syndrome by RFCA via subxiphoid epicardial access in Brazil, demonstrating promising outcomes in medium-term observation. Since the intervention, the patient´s electrocardiographic monitoring has consistently shown no evidence of the BrP, and importantly, no additional life-threatening arrhythmic events throughout the 24-month clinical follow-up period. Regardless of the adopted strategy, RFCA is a targeted and precise treatment that may provide a safe and effective solution for individuals with BrS. However, the optimal protocol for identifying the ablation targets and best approaches to radiofrequency application remains to be defined. Additionally, there is limited data on long-term follow-up after ablation, and no evidence supports ablation in asymptomatic patients. Further research is needed to clarify the precise role of radiofrequency catheter ablation in managing patients with BrS.
